# Assembly of strongly phosphorescent hetero-bimetallic and -trimetallic [2]catenane structures based on a coinage metal alkynyl system†
†Electronic supplementary information (ESI) available: Experimental details. CCDC 1499658 (**1**), 1499630 (**2**), 1499654 (**3**), 1554172 (**4**), 1554173 (**5**), 1554171 (**6**), and 1554174 (**7**). For ESI and crystallographic data in CIF or other electronic format see DOI: 10.1039/c7sc03529b


**DOI:** 10.1039/c7sc03529b

**Published:** 2017-10-04

**Authors:** Xiao-Yong Chang, Guang-Tao Xu, Bei Cao, Juan-Yu Wang, Jie-Sheng Huang, Chi-Ming Che

**Affiliations:** a State Key Laboratory of Synthetic Chemistry , Institute of Molecular Functional Materials , Department of Chemistry , The University of Hong Kong , Pokfulam Road , Hong Kong , China . Email: jshuang@hku.hk ; Email: cmche@hku.hk; b HKU Shenzhen Institute of Research and Innovation , Shenzhen 518053 , China

## Abstract

Homo-metallic metal alkynyl complexes exhibit interesting catenane structures, but their hetero-metallic catenane counterparts are under-developed. In this work, we report rare examples of bimetallic Au–Cu (DtbpC≡C^–^ ligand; Dtbp = 3,5-di-*tert*-butylphenyl), Au–Ag (^*t*^BuC≡C^–^ ligand), and Au–Cu, Au–Ag (C6-FluoC≡C^–^ ligand; C6-Fluo = 9,9-dihexyl-9*H*-fluoren-2-yl) complexes as well as a trimetallic Au–Ag–Cu (C6-FluoC≡C^–^ ligand) complex, which feature [2]catenane structures. The formation of the [2]catenane structure is significantly affected by the coinage metal ion(s) and change of the structure of the alkynyl ligand. These hetero-metallic [2]catenane structures are strongly luminescent with tunable emission *λ*_max_ from 503 to 595 nm and *Φ* values up to 0.83.

## Abstract 2

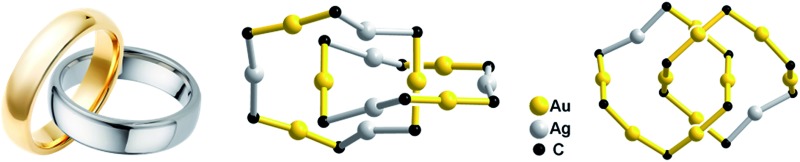
Strongly phosphorescent hetero-metallic [2]catenanes, including bimetallic (RC≡C)_12_Au_6_M_6_ (M = Ag or Cu), (RC≡C)_12_Au_10_Ag_2_ and trimetallic (RC≡C)_12_Au_6_Cu_*n*_Ag_6–*n*_, were obtained.

## Introduction

**Fig. 1:**
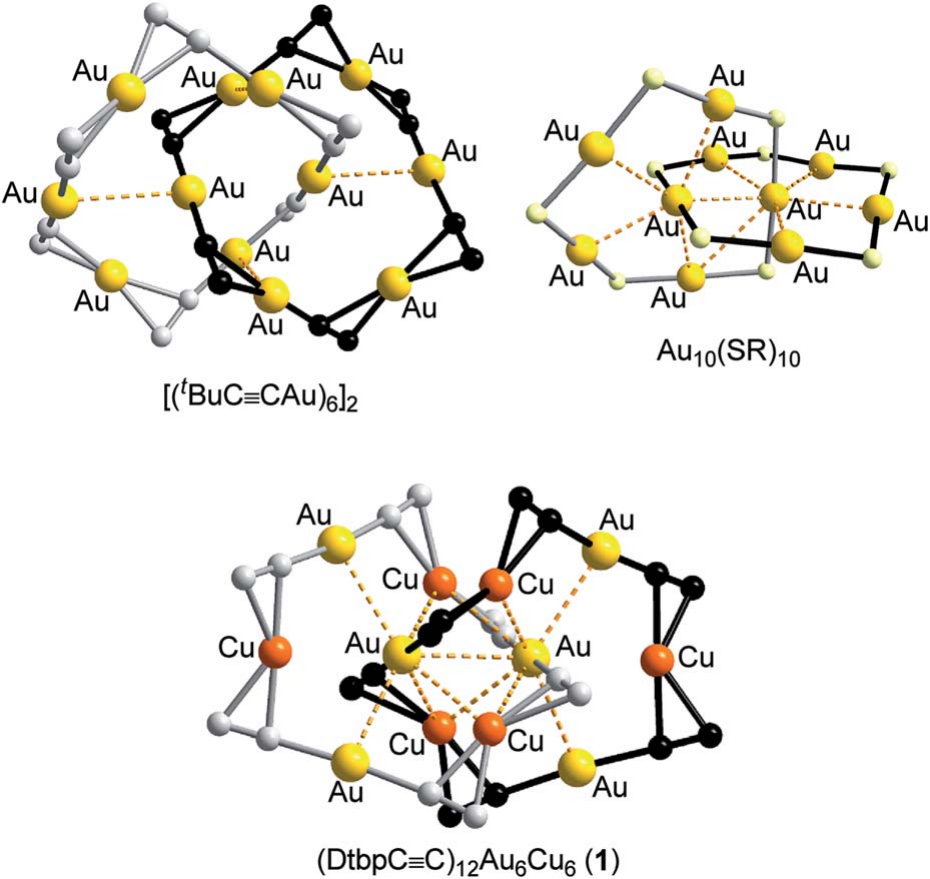
Upper: Literature examples of homo-metallic [2]catenanes. Lower: Hetero-metallic [2]catenane **1**, an example of five hetero-metallic [2]catenanes reported in this work. The peripheral groups (^*t*^Bu, R or Dtbp) are not shown. The metallophilic interactions are depicted as dashed lines.

The construction of intriguing types of interpenetrated structures, including catenanes, continues to be an active area in supramolecular science. Various strategies, including π–π stacking, hydrogen bonding, metal templating, and hydrophobic interactions, have been developed to direct the assembly of interpenetrated structures.^1^ Closed-shell metallophilic interactions could also be appealing driving forces for the formation of catenanes, as demonstrated by the [2]catenane structures of [(^*t*^BuC≡CAu)_6_]_2_,^2^ (RC≡CAu)_10_,^3^ and Au_*n*_(SR)_*n*_ (*n* = 10,^4^ 11,^5^ and 12 (ref. 4)) which feature Au^I^–Au^I^ interactions (Au–Au 2.88–3.30 Å, *e.g.*Fig. 1, upper). We previously reported a [3]catenane structure of (^*t*^BuC≡CCu)_20_.^6^ This type of catenane, first reported by Mingos and co-workers,^2^ is based on homoleptic homo-metallic alkynyl or thiolate complexes. Puddephatt and co-workers reported heteroleptic Au^I^-alkynyl/phosphine complexes adopting [2]catenane structures, which also feature Au^I^–Au^I^ interactions.^7^ The quest remains for hetero-metallic catenanes based on a homoleptic metal alkynyl system.

**Fig. 2:**
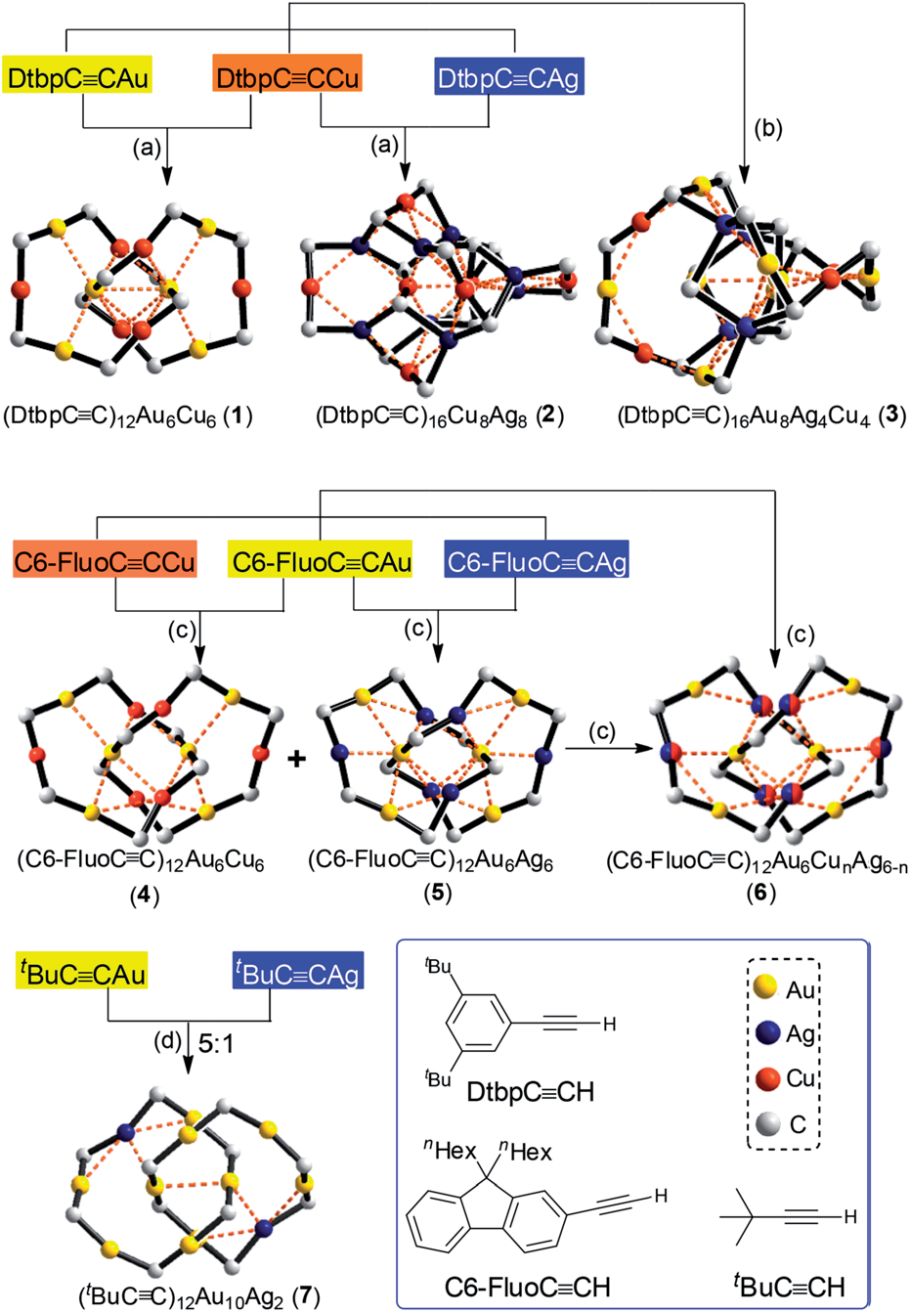
The synthetic scheme and coordination cores of hetero-metallic coinage metal complexes **1–7**. The metallophilic interactions are depicted as dashed lines; for each alkynyl ligand, only the α carbon atom is shown. Reagents and conditions: (a) CH_2_Cl_2_/MeCN; (b) chlorobenzene; (c) toluene/MeCN; (d) CH_2_Cl_2_/Et_3_N.

An important feature of Au^I^-alkynyl catenanes is the presence of two linear RC≡C–Au–C≡CR units in the locking center.^2^,^3^ These units function as a template to facilitate the formation of the first ring and as a building block for the second ring. In view of the RC≡C–M–C≡CR species commonly seen in the literature,^8^ homoleptic hetero-metallic coinage metal alkynyl complexes might also be suitable candidates for the construction of catenanes. A key issue is the control of the complex size, which is tunable by adjusting the bulkiness and/or substitution pattern of the alkynyl ligand.^9^ However, the design of new structures with specific configurations is hampered by the complexity and limited understanding of the structures of such complexes,8a,^10^,^11^ particularly for trimetallic ones.10c,d Also, in view of their intriguing phosphorescence and potential materials application,^12^,^13^ the exploration of new structures of hetero-metallic alkynyl complexes with high stability could be rewarding. Based on our previous work on a Cu^I^–alkynyl system,^9^ we employed bulky alkynyl ligands RC≡C^–^ (R = 3,5-di-*tert*-butylphenyl (Dtbp), 9,9-dihexyl-9*H*-fluoren-2-yl (C6-Fluo), or ^*t*^Bu) to construct novel assemblies of hetero-metallic alkynyl complexes. Herein, we described the formation of five hetero-metallic alkynyl [2]catenanes (including bimetallic **1**, **4**, **5** and **7**, and trimetallic **6**, Fig. 1 and 2) by the self-assembly of homoleptic coinage metal alkynyl systems. As revealed by the structures of these complexes and two other hetero-metallic complexes **2** and **3** (Fig. 2), the proper combination of coinage metal ions and alkynyl ligands is crucial to the formation of the catenane structure.

## Results and discussion

The bimetallic complexes **1**, **2**, **4**, **5** and **7** were prepared by mixing two homoleptic metal complexes in a 1 : 1 or 5 : 1 (for **7**) molar ratio, and the trimetallic complexes **3** and **6** were prepared by mixing the homoleptic gold, silver and copper alkynyl complexes in a 2 : 1 : 1 molar ratio or by mixing the hetero-metallic Au–Cu and Au–Ag alkynyl complexes in a 1 : 1 molar ratio. Complexes **1–3** were also accessible from the reactions of alkynes with Au(SMe_2_)Cl, AgOTf and/or [Cu(MeCN)_4_]PF_6_ (2 : 1 : 1 for **1** and **2** and 3 : 1 : 1 : 1 for **3**) in the presence of Et_3_N (yields: 27–78%). X-ray diffraction-quality crystals of **1–7** were obtained by the slow evaporation of the CH_2_Cl_2_/MeCN, chlorobenzene or toluene/MeCN solutions and their structures were determined by X-ray crystallography (Tables S1 and S2 in the ESI†).

**Fig. 3:**
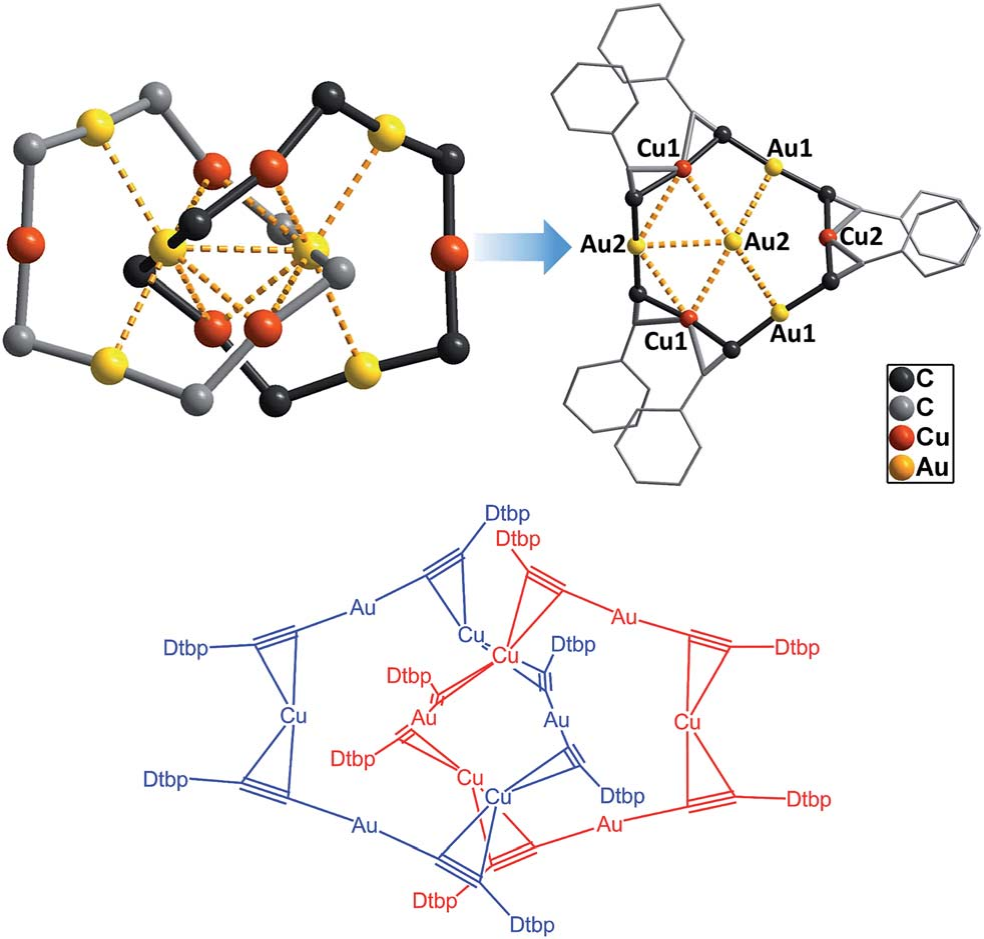
The [2]catenane structure of **1**. Upper left: the coordination core. Upper right: one of the two interlocked (DtbpC≡C)_6_Au_3_Cu_3_ rings. The ^*t*^Bu groups and hydrogen atoms are omitted for clarity, and the metallophilic interactions are depicted as dashed lines. Lower: a wire frame representation with the omission of the metallophilic interactions.

**Fig. 4:**
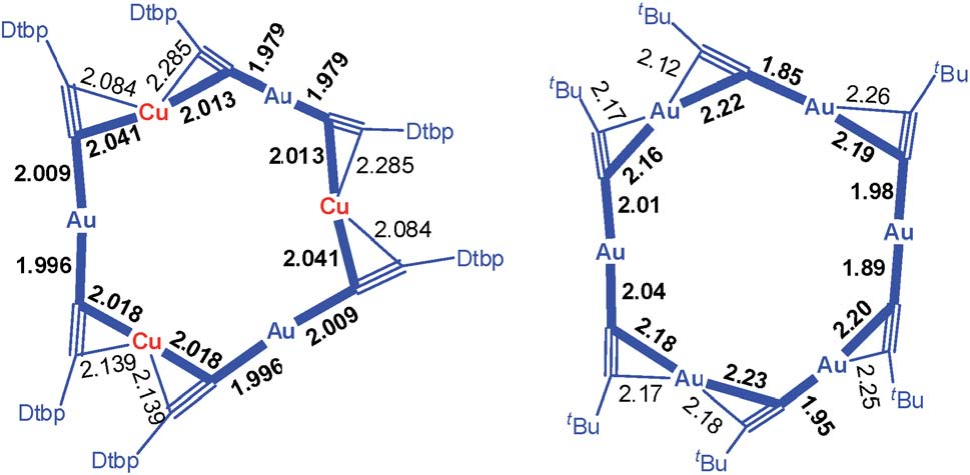
The comparison of the M–C distances in the [2]catenanes **1** (left) and [(^*t*^BuC≡CAu)_6_]_2_ (right). Only one ring is depicted in each case; the metallophilic interactions are not shown.

The Au–Cu complex **1**, with a formula of (DtbpC≡C)_12_Au_6_Cu_6_, has a crystallographic *D*_2_ symmetry and features two twisted (DtbpC≡C)_6_Au_3_Cu_3_ rings that are interlocked to form a [2]catenane structure (Fig. 3). Each ring is composed of three linear DtbpC≡C–Au–C≡CDtbp units and three Cu ions with relatively weak Au–Cu (2.9632(10) Å) and Au–Au (2.9625(3)–3.1497(8) Å) interactions (*cf.* the sums of the metallic radii: Au–Cu 2.72 Å and Au–Au 2.88 Å (ref. 14)), which are comparable to the metallophilic interactions in the well documented Au^I^-alkynyl and -thiolate [2]catenanes (Au–Au 2.88–3.30 Å).^2^–^5^ The bridging alkynyl groups each coordinate to the Au and Cu ions in the η^1^- and η^2^-modes, respectively. The M–C distances (Au–C 1.979(7)–2.009(6) Å and Cu–C 2.018(7)–2.285(14) Å) are comparable to those in the homo-metallic [2]catenane [(^*t*^BuC≡CAu)_6_]_2_ (Au–C 1.85(4)–2.26(3) Å)^2^ (Fig. 4). These observations make it reasonable to describe **1** as a hetero-metallic [2]catenane. In contrast, the PhC≡C^–^ counterpart of **1**, (PhC≡C)_12_Au_6_Cu_6_, adopts a non-catenane structure in which the six Au^I^ ions are co-planar and the six Cu^I^ ions form a trigonal prism with the PhC≡C^–^ ligands situated on the two opposite sides, rendering the Ph groups (for each side) rather close to each other10*i* and reflecting the unique role of the bulky Dtbp groups of **1** in the assembly/stabilization of the [2]catenane structure of the Au_6_Cu_6_ alkynyl complex. It is probable that the non-catenane structure of (PhC≡C)_12_Au_6_Cu_6_, upon changing its alkynyl ligands to the bulkier and more basic DtbpC≡C^–^ ligands, is destabilized owing to the increased steric hindrance [resulting from the bulkiness of the electron-donating ^*t*^Bu substituents and stronger metal-alkynyl binding (*cf.* Au–C 2.004(6)–2.045(5) Å in (PhC≡C)_12_Au_6_Cu_6_ (ref. 10*i*) *vs.* 1.979(7)–2.009(6) Å in **1**)], and as such the steric hindrance is minimized in the [2]catenane structure of the Au–Cu complex **1**.

**Fig. 5:**
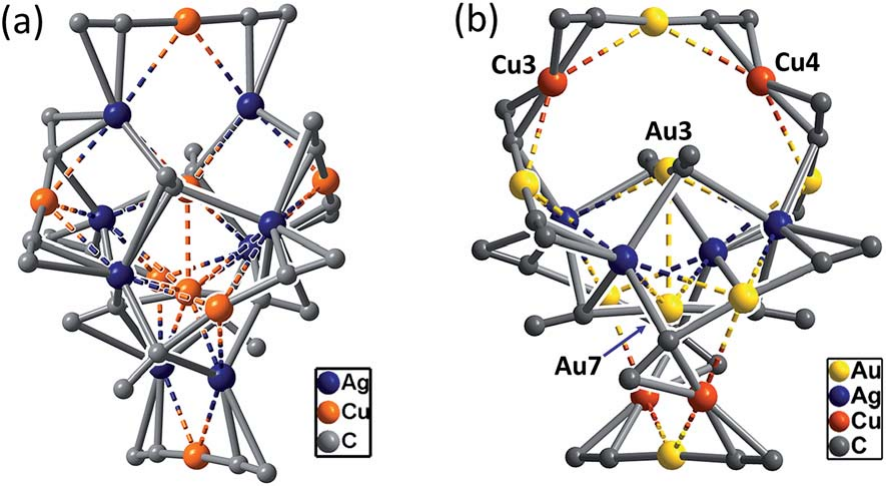
A perspective view of **2** (a) and **3** (b). The Dtbp groups of the alkynyl ligands are omitted for clarity. The metallophilic interactions are depicted as dashed lines.

**Fig. 6:**
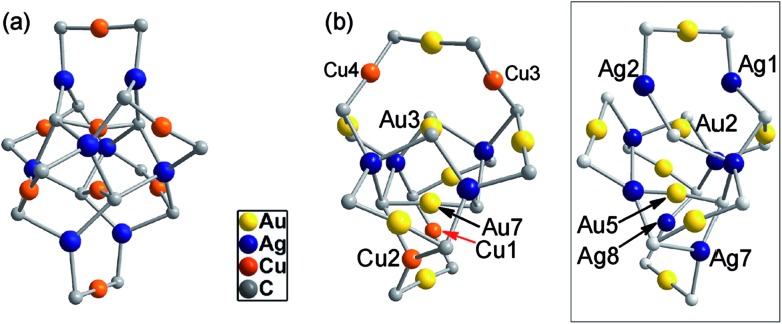
The metallacycle cores in **2** (a) and **3** (b) as compared with that in (DtbpC≡C)_16_Au_8_Ag_8_ (ref. 11*b*) (inset) with the omission of all the metallophilic interactions (for each alkynyl ligand, only the α carbon atom is shown).

By changing Au–Cu to Cu–Ag, but with the same DtbpC≡C^–^ ligand unchanged, a non-catenane complex (DtbpC≡C)_16_Cu_8_Ag_8_ (**2**) was obtained. Complex **2** has a structure with an approximate *S*_4_ symmetry (Fig. 5a, Cu–Ag 2.6824(3)–3.0416(4) Å) and contains a rather complicated metallacycle core (Fig. 6a, Cu–C 1.855(3)–1.889(3) Å, Ag–C 2.236(3)–2.643(2) Å); the topology of its metallophilic interactions is similar to that of the recently reported Au–Ag counterpart (DtbpC≡C)_16_Au_8_Ag_8_, also with a non-catenane structure.11*b* In the case of **3**, its structure (Fig. 5b) features a metallacycle core (Au–C 1.950(7)–2.025(9) Å, Cu–C 1.919(8)–2.038(6) Å, Ag–C 2.364(6)–2.618(7) Å) which is markedly different from that formed by simply replacing the four Ag ions of the (DtbpC≡C)_16_Au_8_Ag_8_ molecule11*b* with four Cu ions. As shown in Fig. 6b, compared with (DtbpC≡C)_16_Au_8_Ag_8_, **3** features central Au–Au distance (Au2–Au5 2.9591(9)11*b* ∼0.22 Å longer *vs.* Au3–Au7 3.1825(4) Å) and substantially larger C–M–C (M = Cu3, Cu4) angles (139.4(5)–142.6(4)° (M = Ag1, Ag2)11*b**vs.* 172.4(3)–177.0(4)°).

**Fig. 7:**
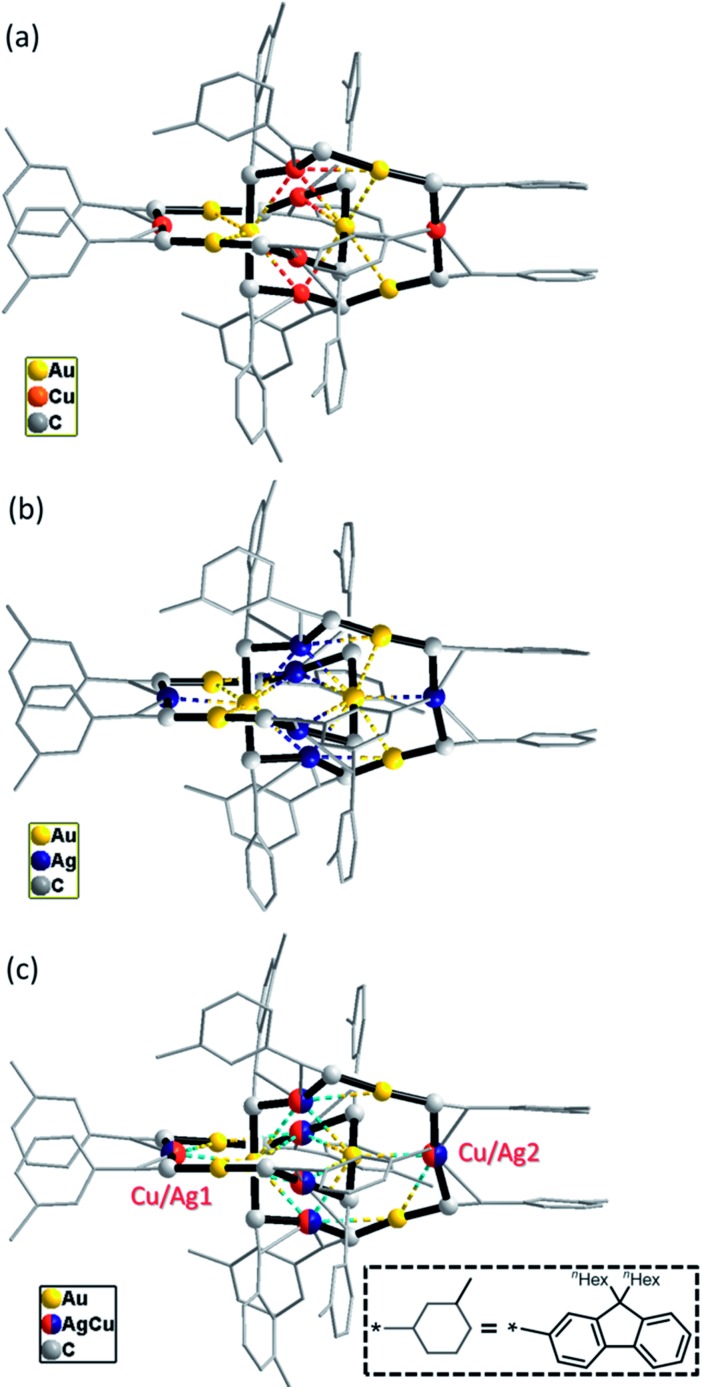
A perspective view of the C6-FluoC≡C^–^ ligand based complexes **4** (a), **5** (b) and **6** (c). All of the hydrogen atoms and parts of the C6-FluoC≡C^–^ ligands are omitted for clarity. The metallophilic interactions are depicted as dashed lines.

Changing the R group of RC≡C^–^ from Dtbp to the bulkier C6-Fluo resulted in the formation of bimetallic (C6-FluoC≡C)_12_Au_6_Cu_6_ (**4**) and (C6-FluoC≡C)_12_Au_6_Ag_6_ (**5**) and trimetallic (C6-FluoC≡C)_12_Au_6_Cu_*n*_Ag_6–*n*_ (**6**), and all of the three complexes adopt a [2]catenane structure (Fig. 7). The arrangement of the six RC≡C–Au–C≡CCR units in **4–6** is similar to that in **1**; the connection of the C6-FluoC≡C–Au–C≡CC6-Fluo units by π-C≡C–Cu/Ag coordination forms the [2]catenane structures. The Cu and Ag atoms in **6** are in substitutional disorder (Fig. 7c): each π-C≡C–M is partially occupied by Cu and Ag atoms, the occupancy of the Ag atom of the outlier positions 1 and 2 (0.77 and 0.62, respectively) is slightly higher (Fig. 7c), and the overall Cu/Ag ratio (3.1 : 2.9) is close to the molar ratio (1 : 1) of **4** and **5** used in the preparation of **6**. The average Cu/Ag–C(α) distance in **6** is 0.14 Å longer than the average Cu–C(α) distance in **4** and 0.12 Å shorter than the average Ag–C(α) distance in **5**, while the difference of the average Au–C(α) distances between **4–6** is <0.03 Å.

**Fig. 8:**
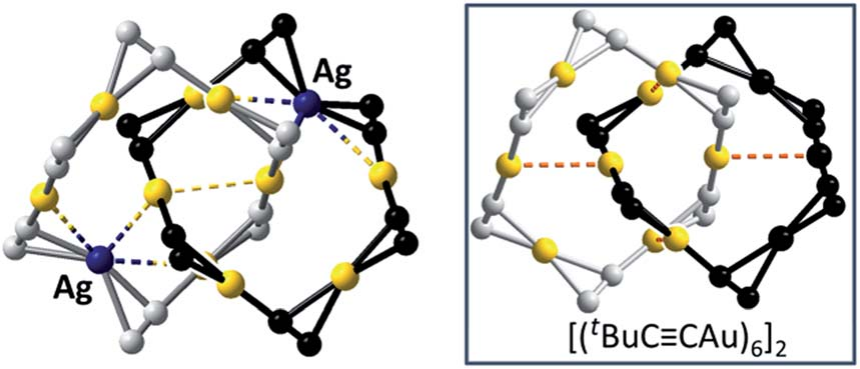
A perspective view of the [2]catenane **7**. Inset: a perspective view of [(^*t*^BuC≡CAu)_6_]_2_ (ref. 2) for comparison. The ^*t*^Bu groups are omitted for clarity. The metallophilic interactions are depicted as dashed lines.

As [2]catenane **6** can be formed by mixing the [2]catenanes **4** and **5** in solution, we also mixed the previously reported [2]catenane [(^*t*^BuC≡CAu)_6_]_2_ (ref. 2) with [^*t*^BuC≡CAg]_*n*_ (molar ratio 5 : 1), and obtained **7**, adopting a [2]catenane structure similar to that of [(^*t*^BuC≡CAu)_6_]_2_ except for the replacement of one bis(η^2^-C≡C) coordinated Au ion in each ring by one Ag ion (Fig. 8).

**Fig. 9:**
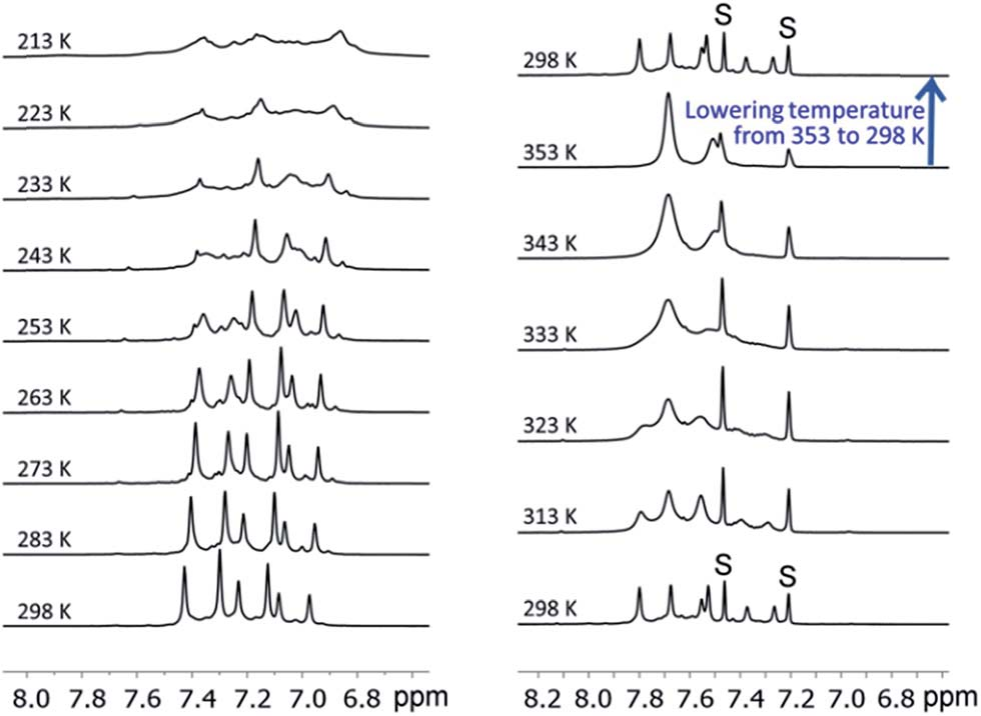
The variable-temperature ^1^H NMR spectra (in the aromatic region) of **1** in CD_2_Cl_2_ (left, 298 K → 213 K) and in 1,2-dichlorobenzene-*d*_4_ (right, 298 K → 353 K → 298 K).

We examined the solution behavior of the hetero-metallic [2]catenanes **1** and **4–7**, which are stable in solution at a concentration >10^–4^ M, using ESI-MS and ^1^H NMR measurements (see the ESI†); the results for **1** are discussed here as examples. The ESI mass spectrum of **1** (∼10^–4^ M) in CH_2_Cl_2_ features a prominent cluster peak at *m*/*z* 4146.4 attributed to [**1** + Na]^+^ (Fig. S1, ESI†), like the observation of cluster peaks at *m*/*z* 5876.4, 6142.2, and 6009.3, which are attributed to [**4** + Na]^+^, [**5** + Na]^+^, and [**6** + Na]^+^ for **4**, **5**, and **6**, respectively. In the ^1^H NMR spectrum of **1** in CD_2_Cl_2_ and 1,2-dichlorobenzene-*d*_4_ (∼10^–2^ M, Fig. 9), three sets of coordinated DtbpC≡C^–^ signals were observed at room temperature (consistent with the *D*_2_ symmetry in the crystal structure of **1**), which were broadened into one set upon increasing the temperature to 353 K and were then recovered by cooling back to room temperature (Fig. 9, upper right). These spectral changes could be associated with the dependence of the metallophilic interactions in **1** on temperature. We further examined the solution of **1** in CD_2_Cl_2_ at room temperature by ^1^H DOSY NMR measurements; the spectrum obtained (Fig. S4, ESI†) reveals that the observed signals of DtbpC≡C^–^ belong to a single complex (diffusion constant *D* = 8.32 × 10^–10^ m^2^ s^–1^), thus providing additional evidence for the purity of **1** in solution.

**Fig. 10:**
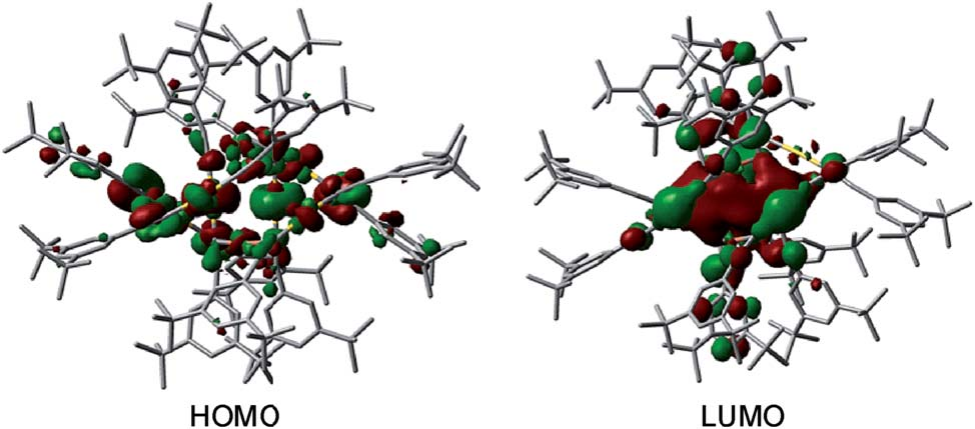
The HOMO and LUMO of **1**. The hydrogen atoms are omitted for clarity. Color code: carbon (grey), gold (gold), and copper (orange).

DFT calculations were performed to examine the electronic details of the hetero-metallic [2]catenanes using **1** as an example. The DFT-optimized geometry of **1** is comparable to that determined by X-ray crystal analysis. For example, the computed structure of **1** features average values of Au–C 1.997 Å, C–Au–C 176.2°, and Cu–C 2.053 Å; these values compare well with the corresponding ones in the crystal structure of **1** (average values: Au–C 1.995(7) Å, C–Au–C 176.9(8)° and Cu–C 2.097(7) Å). To gain insight into why the [2]catenane of (DtbpC≡C)_12_Au_6_M_6_ was obtained for M = Cu (**1**) but not for M = Ag, we attempted to perform DFT optimization of (DtbpC≡C)_12_Au_6_Ag_6_ with a hypothetical similar [2]catenane structure, which did not converge. The highest occupied molecular orbital (HOMO) and lowest unoccupied molecular orbital (LUMO) of **1** are depicted in Fig. 10. The HOMO is mainly localized on the 5d_*z*^2^_ orbitals of the two Au atoms in the locking center of the [2]catenane structure, while the LUMO is distributed on the empty 6p orbitals of the same two Au atoms.

**Fig. 11:**
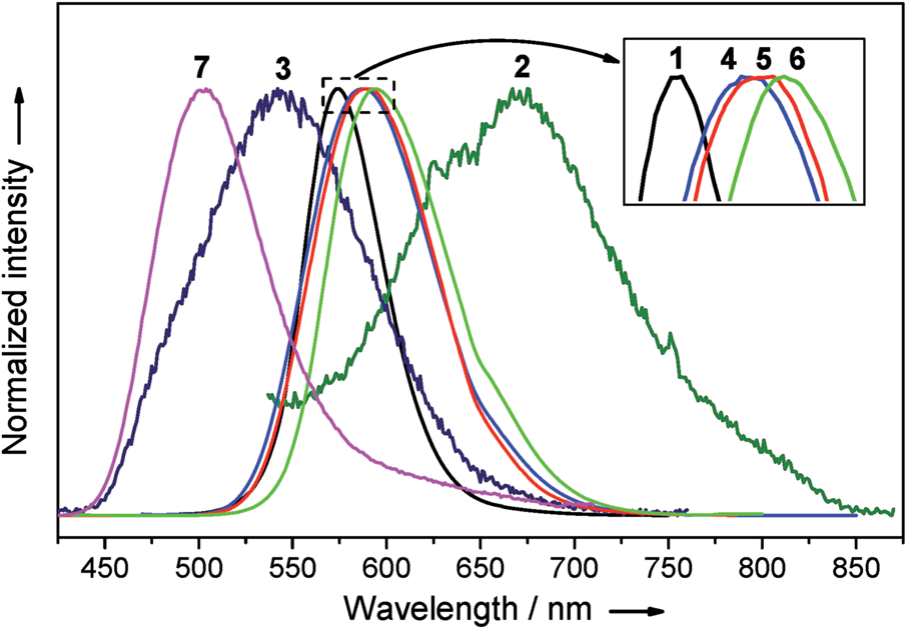
The emission spectra of **1–7** in the solid state.

Complexes **1–7** are emissive in the solid state (Fig. 11). In view of their structural fluxional behavior in solution, their photo-physical properties in solution were not included in this study. The [2]catenanes **1** and **4–6** exhibit moderate yellow to strong orange emissions in the solid state (*Φ* = 0.37–0.83). Changing the ligand from DtbpC≡C^–^ to C6-FluoC≡C^–^ resulted in a bathochromic shift in emission energy and a significant improvement in the quantum yield. Notably, the emission energy and efficiency show only minor variation with the metal compositions of **4–6** (*λ*_max_ = 588–595 nm, *Φ* = 0.71–0.83, and *τ* = 0.7–1.0 μs). The comparison of the emission spectra of (DtbpC≡C)_12_Au_8_Ag_8_ (*λ*_max_ = 489 nm (ref. 11*b*)) and **3** reveals that the replacement of the four Ag ions by four Cu ions resulted in a broader and red-shifted emission band (*λ*_max_ = 542 nm). The excitation of **7** in the solid state gave a strong green emission at *λ*_max_ 503 nm with a tail up to 710 nm (*Φ =* 0.82). The wide span in emission energy (*λ*_max_ from 503 to 595 nm) and high solid state emission quantum yields highlight the prospect of hetero-metallic [2]catenanes based on a coinage metal alkynyl system as useful photo-functional molecular materials.

The use of the C6-FluoC≡C^–^ ligand to result in the assembly of hetero-bimetallic and hetero-trimetallic [2]catenanes (RC≡C)_12_Au_6_M_6_ (M = Cu **4** and Ag **5**) and (RC≡C)_12_Au_6_Cu_*n*_Ag_6–*n*_ (**6**) is remarkable. As **4–6** are nearly isostructural, it appears that their C6-FluoC≡C^–^ ligands dominate the intermolecular interactions, with the effect of the Ag and Cu ions being minor in these cases. As revealed by the crystal structures of **4** and **5**, replacing the Cu ions with Ag ions slightly expands the metallacycle core owing to the longer Ag–C than Cu–C distances (Fig. S5, ESI†). The expansion of the metallacycle core would reduce the repulsion between the peripheral alkynyl ligands and increase the tendency of the complex to re-assemble to higher nuclearity species. The bulky C6-FluoC≡C^–^ ligand with flexible C_6_-alkyl chains is likely to restrict such tendency. For the complexes of the DtbpC≡C^–^ ligand, which is sterically less demanding and relatively rigid, the replacement of the Cu ions by Ag ions leads to a core enlargement from M_12_ (**1**) to M_16_ (**3**). On the other hand, an Ag ion, compared with a Cu ion, is a stronger Lewis acid and is inclined to form weak interactions with more alkynyl ligands (*cf.***2** and **3** in Fig. 5); the extra π-C≡C–Ag interactions may distort the ring unit and then break the [2]catenane structure. Moreover, the preference of Au^I^ for a linear two-coordinate configuration should also play an important role in the assembly of the hetero-metallic [2]catenanes in view of the core enlargement from M_12_ (**1**) to M_16_ (**2**), upon replacing the Au ions with Ag ions, and the presence of linear RC≡C–Au^I^–C≡CR units in all of the [2]catenanes **1**, **4–6**, and **7**.

## Conclusions

We have prepared and structurally characterized five hetero-metallic [2]catenanes based on coinage metal alkynyl complexes, including bimetallic Au–Cu and Au–Ag complexes and a trimetallic Au–Cu–Ag complex, by employing bulky DtbpC≡C^–^, C6-FluoC≡C^–^, and ^*t*^BuC≡C^–^ ligands. The structure of the trimetallic [2]catenane **6** is analogous to its corresponding Au–Ag bimetallic [2]catenanes with some of the Ag atoms replaced by Cu atoms; mixing the Au–Cu and Au–Ag complexes is a feasible and efficient method to prepare the trimetallic complex. The formation of [2]catenanes relies upon a delicate balance between various intermolecular interactions. The structural characterization of **1–7** provides useful insight into a better understanding of hetero-metallic coinage metal alkynyl complexes.

## Conflicts of interest

There are no conflicts to declare.

## Supplementary Material

Supplementary information

Crystal structure data
