# Supplementary material for: Assembly of strongly phosphorescent hetero-bimetallic and -trimetallic [2]catenane structures based on a coinage metal alkynyl system
Source: Chem Sci. 2017 Oct 4;8(11):7815–20. doi: 10.1039/c7sc03529b (PMC5694978; doi:10.1039/c7sc03529b)
Supplement: Supplementary file 1 [file SC-008-C7SC03529B-s001.pdf]

Electronic Supplementary Material (ESI) for Chemical Science.
This journal is © The Royal Society of Chemistry 2017

Electronic Supplementary Information

Table of Contents

Materials and instrumentation 2
Syntheses of **1**–**7** 2
Crystal structure determination 4
Computational studies 5
Supporting Tables and Figures 6
Table S1 Crystallographic data for **1**–**4** 6
Table S2 Crystallographic data for **5**–**7** 7
Table S3 Selected M–M and M–C(α) distances in **1**–**7**. 8
Table S4 Photophysical data of **1**–**7** 12
Fig. S1 ESI-MS spectrum of **1** in CH_2Cl_2/MeOH (c ≈ 10^{-4} M) 12
Fig. S2 ^{1}H NMR spectrum of **1** in 1,2-dichlorobenzene-d_4 at 298 K 13
Fig. S3 ^{1}H-^{1}H COSY NMR spectrum (in aromatic region) of **1** in CD_2Cl_2 at 298 K. 13
Fig. S4 ^{1}H DOSY NMR spectrum of **1** in CD_2Cl_2 at 298 K 14
Fig. S5 Comparison of metallacycle core in **4** and **5** 14
Fig. S6 ESI-MS spectrum of **3** in CH_2Cl_2. 15
Fig. S7 Variable-temperature ^{1}H NMR spectra of **5** in $CDCl_3$. 15
References 16

Materials and instrumentation

All starting materials were purchased from commercial sources and used as received unless stated otherwise. The solvents used for synthesis were of analytical grade. The compounds 3,5-di-*tert*-butylphenylacetylene,[1] $[^t\mathrm{BuC{\equiv}CAu}]_{12}$ [2] and $[^t\mathrm{BuC{\equiv}CAg}]_n$ [2] were prepared according to the literature procedures.

^{1}H NMR and ^{1}H-^{1}H COSY NMR spectra were recorded on a Bruker Avance 400 or 500 DRX FT-NMR spectrometer. Elemental analyses were performed on a Flash EA 1112 elemental analyzer at the Institute of Chemistry, Chinese Academy of Sciences, Beijing. Electrospray-ionization (ESI) mass spectra were recorded on a Waters Q-TOF Premier mass spectrometer. Emission lifetime measurements were performed on a Quanta Ray DCR-3 pulsed Nd:YAG laser system (pulse output 355 nm, 8 ns). The photocurrent signal was recorded by a Tektronix Inc. Model 2430 digital oscilloscope and processed using Origin 8.5 for exponential fit. Emission spectra were performed on a Spex Fluorolog Model 1168 fluorescence spectrometer.

Syntheses of 1–7

DtbpC≡CAu: Diisopropylamine (1 mL) was added to a solution of 3,5-di-*tert*-butylphenylacetylene (214 mg, 1 mmol) and $Au(SMe_2)Cl$ (295 mg, 1 mmol) in dichloromethane (10 mL) with stirring. White precipitate was observed at the start and disappeared with addition of more diisopropylamine. Five minutes later, the clear solution was concentrated and the white precipitate was collected and washed with methanol to yield **DtbpC≡CAu** as a white solid (399 mg, yield: 78%). Elemental analysis: calculated (%) for $C_{16}H_{21}Au{\cdot}C_6H_{15}N$: C 51.66, H 7.09, N 2.74; found: C 51.26, H 7.14, N 2.76.

DtbpC≡CAg: Triethylamine (0.5 mL) was added to a solution of 3,5-di-*tert*-butylphenylacetylene (214 mg, 1 mmol) and AgOTf (258 mg, 1 mmol) in methanol (5 mL), giving a white precipitate immediately. After stirring for 5 min, the precipitate was collected by filtration and dried in vacuum to yield **DtbpC≡CAg** as a white solid (216 mg, yield: 67%). Elemental analysis: calculated (%) for $C_{16}H_{21}Ag$: C 59.83, H 6.59; found: C 59.69, H 6.67.

DtbpC≡CCu: Triethylamine (1 mL) was added to a solution of 3,5-di-*tert*-butylphenylacetylene (214 mg, 1 mmol) and $[Cu(MeCN)_4]PF_6$ (372 mg, 1 mmol) in dichloromethane (5 mL), giving an orange precipitate immediately. After stirring for 10 min, the precipitate was collected by filtration and dried in vacuum to yield **DtbpC≡CCu** as an orange solid (254 mg, yield: 92%). **DtbpC≡CCu** has a poor solubility in common solvents. Elemental analysis: calculated (%) for $C_{16}H_{21}Cu{\cdot}0.15CH_2Cl_2$: C 66.97, H 7.41; found: C 67.00, H 7.31.

C6-FluoC≡CAu: Triethylamine (0.5 mL) was added to a solution of 9,9-dihexyl-9*H*-fluoren-2-ylacetylene (179 mg, 0.5 mmol) and $Au(SMe_2)Cl$ (198 mg, 0.5 mmol) in dichloromethane (5 mL) to yield a clear yellow solution. After stirring for 30 min, the solution was poured into a beaker containing acetonitrile (50 mL) and the yellow precipitate was collected by

filtration and dried in vacuum to yield **C6-FluoC≡CAu** as a yellow solid (210 mg, yield: 76%). Elemental analysis: calculated (%) for $C_{27}H_{33}Au$: C 58.48, H 6.00; found: C 58.34, H 6.00.

C6-FluoC≡CAg: The procedure is similar to that for the preparation of **DtbpC≡CAg** except that 9,9-dihexyl-9*H*-fluoren-2-ylacetylene (358 mg, 1 mmol) was used instead of 3,5-di-*tert*-butylphenylacetylene, yielding **C6-FluoC≡CAg** as an off-white solid. (405 mg, yield: 87%). Elemental analysis: calculated (%) for $C_{27}H_{33}Ag$: C 69.68, H 7.15; found: C 69.04, H 7.32.

C6-FluoC≡CCu: Triethylamine (0.5 mL) was added to a solution of 9,9-dihexyl-9*H*-fluoren-2-ylacetylene (179 mg, 0.5 mmol) and $[Cu(MeCN)_4]PF_6$ (223 mg, 0.6 mmol) in acetonitrile/dichloromethane (20 mL) in argon atmosphere, giving an orange precipitate immediately. After stirring for 30 min, the precipitate was collected by filtration and dried in vacuum to yield **C6-FluoC≡CCu** as an orange solid (193 mg, yield: 92%). Elemental analysis: calculated (%) for $C_{27}H_{33}Cu{\cdot}0.5CH_2Cl_2$: C 71.25, H 7.39; found: C 71.48, H 7.56.

1: *Method A:* A mixture of **DtbpC≡CCu** (28 mg, 0.1 mmol) and **DtbpC≡CAu** (50 mg, 0.1 mmol) in dichloromethane (10 mL) was stirred overnight at room temperature to yield a clear yellow solution. The solvent was evaporated in vacuum, and the residue was washed with methanol to give **1** as a yellow solid (63 mg, yield 92%). *Method B*: triethylamine (1 mL) was added to a mixture of 3,5-di-*tert*-butylphenylacetylene (214 mg, 1 mmol), $[Cu(MeCN)_4]PF_6$ (186 mg, 0.5 mmol) and $Au(SMe_2)Cl$ (148 mg, 0.5 mmol) in dichloromethane (15 mL). The light yellow solution was stirred for 1 h under N_2 atmosphere at room temperature. Slow addition of methanol (25 mL) afforded **1** as a yellow solid (268 mg, yield: 78%). ^1H NMR (400 MHz, $[D_4]$1,2-dichlorobenzene): δ ppm 7.80 (s, 8H), 7.68 (s, 8H), 7.55 (s, 4H), 7.53 (s, 8H), 7.38 (s, 4H), 7.27 (s, 4H), 1.38 (s, 72H), 1.34 (s, 72H), 1.02 (s, 72H); ESI-MS: *m/z* 4146.4 ($[\mathbf{1} + Na]^+$); elemental analysis: calculated (%) for $C_{192}H_{252}Au_6Cu_6$: C 55.93, H 6.16; found: C 56.32, H 6.36.

2: *Method A:* A mixture of **DtbpC≡CCu** (28 mg, 0.1 mmol) and **DtbpC≡CAg** (32 mg, 0.1 mmol) in dichloromethane (20 mL) was stirred overnight at room temperature to yield a clear yellow solution. Slow evaporation of the solvent in air gave **2** as a yellow solid (41 mg, yield: 68%). *Method B*: Triethylamine (1 mL) was added to a solution of AgOTf (129 mg, 0.5 mmol) and 3,5-di-*tert*-butylphenylacetylene (214 mg, 1 mmol) in methanol (5 mL), giving a white precipitate immediately. Then a solution of $[Cu(MeCN)_4]PF_6$ (186 mg, 0.5 mmol) in dichloromethane (5 mL) was added and a yellow precipitate was formed. After stirring at room temperature for 2 h, the mixture was filtered and the yellow solid was washed with methanol and collected as crude product. Recrystallization of the crude product in dichloromethane gave **2** as a yellow solid (191 mg, yield: 64%). ESI-MS: *m/z* 4808.3 ($[\mathbf{2} + Na]^+$); elemental analysis: calculated (%) for $C_{256}H_{336}Ag_8Cu_8$: C 64.26; H 7.08; found: C 63.88, H 7.09.

3: *Method A*: A mixture of **DtbpC≡CCu** (14 mg, 0.05 mmol), **DtbpC≡CAg** (16 mg, 0.05 mmol) and **DtbpC≡CAu** (50 mg, 0.1 mmol) in chlorobenzene (4 mL) was stirred overnight at room temperature to yield a yellow solution. Filtration and slow evaporation gave **3** as a yellow solid (45 mg, yield 61%). *Method B*: Slow evaporation of a solution of **1** (8.2 mg 0.002 mmol) and $(DtbpC{\equiv}C)_{16}Au_8Ag_8$ (11.6 mg, 0.002 mmol) in chlorobenzene gave **3** as light yellow crystals (14.2 mg, yield 72%). *Method C*: To a solution of 3,5-di-*tert*-butylphenylacetylene (321 mg, 1.5 mmol)

in methanol (5 mL) was added AgOTf (129 mg, 0.5 mmol in 2 mL MeOH), Et_3N (1 mL), $[Cu(MeCN)_4]PF_6$ (186 mg, 0.5 mmol in 5 mL CH_2Cl_2) and $Au(SMe_2)Cl$ (148 mg, 0.5 mmol in 5 mL CH_2Cl_2) in turn. A yellow precipitate was observed immediately, and the reaction mixture was stirred for 2 h at room temperature. Filtration and recrystallization from chlorobenzene afforded **3** as light yellow crystals (96 mg, yield: 27%). ESI-MS: *m/z* 5737.4 ($[\mathbf{3} + Cu]^+$); elemental analysis: calculated (%) for $C_{256}H_{336}Au_8Ag_4Cu_4$: C 54.18; H 5.97; found: C 53.86, H 5.96.

4: Slow diffusion of acetonitrile (4 mL) to a solution of **C6-FluoC≡CCu** (10 mg, 0.024 mmol) and **C6-FluoC≡CAu** (11 mg, 0.02 mmol) in toluene (0.5 mL) yielded **4** as orange crystals (8.3 mg, yield: 43%). ^1H NMR (400 MHz, CD_2Cl_2) δ 7.61 (s, 12H), 7.57 (d, *J* = 5.2 Hz, 12H), 7.40–7.23 (m, 48H), 7.17 (d, *J* = 7.5 Hz, 12H), 1.93 (t, *J* = 7.9 Hz, 48H), 1.15–0.92 (m, 144H), 0.73 (t, *J* = 6.9 Hz, 72H), 0.68–0.49 (m, 48H); ESI-MS: *m/z* 5876.4 ($[\mathbf{4} + Na]^+$); elemental analysis: calculated (%) for $C_{324}H_{396}Au_6Cu_6$: C 66.48, H 6.82; found: C 66.10, H 6.92.

5: Slow diffusion of acetonitrile (4 mL) to a solution of **C6-FluoC≡CAg** (9.3 mg, 0.02 mmol) and **C6-FluoC≡CAu** (11 mg, 0.02 mmol) in toluene (0.5 mL) yielded **5** as orange crystals (12 mg, yield: 59%). ^1H NMR (400 MHz, CD_2Cl_2) δ 8.57 (s, 3H), 7.76 (s, 6H), 7.70 (d, *J* = 6.1 Hz, 6H), 7.58 (s, 3H), 7.51–7.01 (m, 51H), 6.89 (d, *J* = 12.0 Hz, 6H), 6.80 (d, *J* = 7.5 Hz, 3H), 6.73 (d, *J* = 8.1 Hz, 3H), 6.59 (d, *J* = 7.7 Hz, 3H), 2.95–2.75 (m, 3H), 2.75–2.52 (m, 3H), 2.28–1.75 (m, 42H), 1.51–0.04 (m, 264H); ESI-MS: *m/z* 6142.2 ($[\mathbf{5} + Na]^+$); elemental analysis: calculated (%) for $C_{324}H_{396}Au_6Ag_6$: C 63.59, H 6.52; found: C 63.66, H 6.62.

6: Slow diffusion of acetonitrile (4 mL) to a solution of **C6-FluoC≡CCu** (4.2 mg, 0.01 mmol) **C6-FluoC≡CAg** (4.7 mg, 0.01 mmol) and **C6-FluoC≡CAu** (11 mg, 0.02 mmol) in toluene (0.5 mL) yielded **6** as orange crystals (7.8 mg, yield: 39%). *Method B*: Slow diffusion of acetonitrile (4 mL) to a solution of **4** (11.7 mg, 0.002 mmol) and **5** (12 mg, 0.002 mmol) in toluene (0.5 mL) yielded **6** as orange crystals (15.5 mg, yield: 67%). ^1H NMR (400 MHz, CD_2Cl_2) δ 8.82–8.24 (m, 3H), 7.87–7.75 (m, 6H), 7.75–7.65 (m, 6H), 7.65–7.45 (m, 3H), 7.44–7.05 (m, 51H), 7.05–6.45 (m, 15H), 3.00–2.74 (m, 3H), 2.74–2.50 (m, 3H), 2.27–1.71 (m, 42H), 1.49–0.15 (m, 264H); ESI-MS: *m/z* 6009.3 ($[\mathbf{6} + Na]^+$); elemental analysis: calculated (%) for $C_{324}H_{396}Au_6Ag_3Cu_3$: C 65.00, H 6.67; found: C 64.90, H 6.74.

7: A mixture of $[^tBuC≡CAu]_{12}$ (50 mg) and $[^tBuC≡CAg]_n$ (7 mg) in CH_2Cl_2/Et_3N (5 mL / 0.5 mL) was heated to reflux for 2 min and then cooled down to room temperature with stirring. After removing solvent in vacuum, the residual was recrystallized in CH_2Cl_2/MeCN to yield **7** as an orange solid (38 mg, yield: 67%). ^1H NMR (500 MHz, CD_2Cl_2) δ 1.51–1.31 (m, 108H). ESI-MS: *m/z* 3077.4 ($[\mathbf{7} – (^tBuC≡C)]^+$); elemental analysis: calculated (%) for $C_{72}H_{108}Au_{10}Ag_2$: C 27.37; H 3.45; found: C 27.64, H 3.52.

Crystal structure determination

Intensity data of capillary-sealed crystal of **1** were collected at 220 K on a Bruker D8 Venture diffractometer with IμS Mo microfocus source. Crystals of **2**–**7** were quickly mounted in a glass fiber and measured at a temperature of 100 K (**2**, **3** and **7**) or 200 K (**4**–**6**); X-ray diffraction data

were collected on a Bruker Proteum X8 diffractometer with monochromated Cu-Kα radiation or Bruker D8 Venture diffractometer with metaljet Ga-Kα radiation (**5**). The Proteum2 program package was used for cell refinement and data reduction.[3] All structures were solved by direct methods using SHELXT and refined by full-matrix least-squares on $|F^2|$ algorithm (SHELXL)[4] using Olex2 program.[5] Some solvent molecules in the crystal structure of **2** and **4**–**6** were omitted using SQUEEZE routing of PLATON program as they were highly disordered and could not be resolved unambiguously. Crystallographic data for **1**–**4** and **5**–**7** are given in Tables S1 and S2, respectively.

Computational studies

Gaussian 09 packages[6] was used to optimized the structure of **1** at the PBE level[7, 8] of density functional theory (DFT); the empirical dispersion correction of Grimme's DFT-D3 dispersion was applied.[9] The 6-31G* Pople basis set[10, 11] was chosen to describe C and H atoms and effective core potential (ECP) type basis set SDD was applied on Cu and Au atoms.[12, 13] To further refine the molecular orbital information, the solvent effect was included by the single point calculations for the optimized gas-phase structure with self-consistent reactions field (SCRF) based on the polarizable continuum model (CPCM)[14] in which dichloromethane was the solvent.

Supporting Tables and Figures

Table S1 Crystallographic data for **1**–**4**.

Complex	**1**	**2**·6CH_2Cl_2	**3**·12C_6H_5Cl	**4**
Empirical formula	$C_{192}H_{252}Au_6Cu_6$	$C_{262}H_{348}Ag_8Cl_{12}Cu_8$	$C_{328}H_{396}Ag_4Au_8Cl_{12}Cu_4$	$C_{324}H_{396}Au_6Cu_6$
Formula weight	4122.96	5294.06	7025.20	5853.42
Temperature/K	220	100	100	200.0
Crystal system	orthorhombic	triclinic	triclinic	monoclinic
Space group	Fddd	$P\bar{1}$	$P\bar{1}$	C2/c
a/Å	24.7200(11)	20.1746(12)	20.2863(14)	79.645(3)
b/Å	30.9053(14)	20.4429(12)	25.3095(17)	18.8256(8)
c/Å	52.473(2)	34.225(2)	30.667(2)	50.821(2)
α/°	90	87.8191(17)	88.6930(10)	90
β/°	90	73.8816(16)	83.3350(10)	127.383(2)
γ/°	90	72.2845(16)	77.7460(10)	90
Volume/Å^3	40088(3)	12900.6(13)	15282.7(18)	60548(5)
Z	8	2	2	8
ρ_{calc}g/cm^3	1.366	1.363	1.527	1.284
F(000)	16416.0	5464.0	7008.0	23904.0
Crystal size/mm^3	0.4 × 0.3 × 0.25	0.35 × 0.25 × 0.1	0.3 × 0.22 × 0.16	0.3 × 0.3 × 0.28
Radiation	MoKα (λ = 0.71073)	CuKα (λ = 1.54178)	CuKα (λ = 1.54178)	CuKα (λ = 1.54178)
2Θ range for data collection/°	4.22 to 50.28	2.69 to 135.384	2.9 to 133.752	2.792 to 118.25
Reflections collected	244098 [R_{int} = 0.0631]	300578 [R_{int} = 0.0715]	281877 [R_{int} = 0.1122]	199273 [R_{int} = 0.0587]
Data/restraints/parameters	8933/1323/795	45465/265/2892	53189/577/3342	43364/1340/3369
Goodness-of-fit on F^2	1.054	1.047	1.016	1.045
Final R indexes [I>=2σ (I)]	R_1 = 0.0663, wR_2 = 0.1793	R_1 = 0.0803, wR_2 = 0.2342	R_1 = 0.0896, wR_2 = 0.2441	R_1 = 0.0888, wR_2 = 0.1708
Largest diff. peak/hole / e Å^{-3}	2.09/-1.18	4.48/-1.90	3.20/-5.74	1.79/-1.32
CCDC number	1499658	1499630	1499654	1554172

Table S2 Crystallographic data for **5**–**7**.

Complex	**5**	**6**	**7**
Empirical formula	$C_{324}H_{396}Ag_6Au_6$	$C_{324}H_{396}Ag_{2.88}Au_6Cu_{3.13}$	$C_{72}H_{108}Ag_2Au_{10}$
Formula weight	6119.40	5980.87	3158.98
Temperature/K	200	200	100
Crystal system	monoclinic	monoclinic	monoclinic
Space group	C2/c	C2/c	C2/c
a/Å	80.362(3)	80.200(4)	28.881(3)
b/Å	18.8467(8)	18.8497(10)	13.0310(12)
c/Å	51.284(2)	51.113(3)	25.373(2)
α/°	90	90	90
β/°	128.2992(14)	128.112(2)	117.008(3)
γ/°	90	90	90
Volume/$Å^3$	60956(4)	60796(6)	8507.5(14)
Z	8	8	4
$\rho_{calc}g/cm^3$	1.334	1.307	2.466
F(000)	24768.0	24318.0	5696.0
Crystal size/mm^3	0.3 × 0.26 × 0.26	0.32 × 0.3 × 0.3	0.24 × 0.21 × 0.18
Radiation	GaKα (λ = 1.34139)	CuKα (λ = 1.54178)	CuKα (λ = 1.54178)
2Θ range for data collection/°	4.966 to 104.228	2.8 to 130.24	6.87 to 133.976
Reflections collected	200547 [R_{int} = 0.0500]	306222 [R_{int} = 0.0666]	72045 [R_{int} = 0.0722]
Data/restraints/parameters	50720/1239/3372	51468/1326/3372	7478/181/484
Goodness-of-fit on F^2	1.144	1.056	1.037
Final R indexes [I>=2σ (I)]	R_1 = 0.1052, wR_2 = 0.2023	R_1 = 0.0827, wR_2 = 0.1936	R_1 = 0.0904, wR_2 = 0.2488
Largest diff. peak/hole / e $Å^{-3}$	2.22/-1.43	2.83/-1.76	7.02/-5.68
CCDC number	1554173	1554171	1554174

Table S3 Selected M–M and M–C(α) distances in **1**–**7**.

Complex **1**			
M–M distances (Å)			
Cu1–Au2	2.9632(10)	Au2–Au1	2.9625(3)
Au2–Au2	3.1497(8)		
M–C(α) distances (Å)			
Cu2–C1	2.018(7)	Au1–C17	2.009(6)
Au1–C1	1.996(6)	Cu1–C33	2.013(13)
Cu1–C17	2.041(7)	Au2–C33	1.979(7)

Complex **2**			
M–M distances (Å)			
Ag1–Cu1	2.7763(5)	Ag5–Cu5	2.9898(5)
Ag1–Cu2	2.7383(4)	Ag5–Cu6	2.8376(5)
Ag1–Cu7	2.7369(5)	Ag6–Cu4	2.8947(4)
Ag2–Cu2	2.8788(3)	Ag6–Cu6	2.8894(4)
Ag2–Cu3	2.8958(5)	Ag6–Cu7	2.8186(4)
Ag2–Cu6	2.9354(4)	Ag7–Cu2	2.9082(5)
Ag3–Cu3	2.8517(4)	Ag7–Cu7	2.8266(4)
Ag3–Cu4	2.9191(4)	Ag7–Cu8	2.9319(4)
Ag3–Cu8	3.0416(4)	Ag8–Cu3	2.7178(5)
Ag4–Cu1	2.8737(5)	Ag8–Cu5	2.7588(4)
Ag4–Cu4	2.7424(4)	Ag8–Cu8	2.6930(4)
Ag4–Cu7	2.6880(4)	Cu3–Cu7	2.5914(5)
Ag5–Cu3	2.6824(3)		
M–C(α) distances (Å)			
Cu1–C1	1.865(3)	Ag2–C209	2.538(2)
Cu1–C17	1.872(3)	Ag3–C81	2.615(2)
Cu2–C33	1.866(2)	Ag3–C113	2.331(2)
Cu2–C49	1.866(2)	Ag3–C193	2.516(2)
Cu3–C65	1.862(2)	Ag3–C225	2.352(2)
Cu3–C81	1.881(2)	Ag4–C1	2.236(3)
Cu4–C97	1.879(3)	Ag4–C97	2.424(2)
Cu4–C113	1.855(3)	Ag4–C193	2.502(3)
Cu5–C129	1.876(3)	Ag5–C81	2.478(2)
Cu5–C145	1.889(3)	Ag5–C129	2.269(3)
Cu6–C161	1.869(2)	Ag5–C177	2.437(3)
Cu6–C177	1.857(2)	Ag6–C81	2.561(2)
Cu7–C193	1.865(2)	Ag6–C97	2.337(2)
Cu7–C209	1.864(3)	Ag6–C161	2.328(2)
Cu8–C225	1.862(2)	Ag6–C209	2.643(2)
Cu8–C241	1.857(2)	Ag7–C33	2.337(3)
Ag1–C17	2.254(3)	Ag7–C65	2.4891(19)
Ag1–C33	2.412(3)	Ag7–C193	2.607(2)
Ag1–C209	2.454(2)	Ag7–C241	2.364(3)
Ag2–C65	2.634(3)	Ag8–C145	2.254(2)
Ag2–C177	2.3300(19)	Ag8–C225	2.461(2)
Ag2–C49	2.356(2)	Ag8–C65	2.480(3)

Complex **3**

M–M distances (Å)

Au1–Cu1	2.840(2)	Au5–Cu3	2.8581(17)
Au1–Cu2	2.9360(12)	Au5–Cu4	2.787(2)
Au2–Ag1	3.0325(6)	Au6–Ag1	3.0066(6)
Au2–Ag4	2.9666(6)	Au6–Ag3	2.9607(6)
Au2–Cu1	2.662(3)	Au6–Cu3	2.6924(16)
Au3–Au7	3.1825(4)	Au7–Ag3	3.0131(6)
Au3–Ag1	2.9387(6)	Au7–Ag4	3.0140(6)
Au3–Ag2	3.0061(6)	Au8–Ag2	2.9556(6)
Au4–Ag2	2.9874(6)	Au8–Ag4	2.9725(7)
Au4–Ag3	3.0052(6)	Au8–Cu4	2.673(2)
Au4–Cu2	2.7487(13)		

M–C(α) distances (Å)

Au1–C1	1.962(9)	Ag2–C97	2.407(6)
Au1–C17	1.994(8)	Ag2–C209	2.542(7)
Au2–C33	1.981(7)	Ag2–C241	2.407(7)
Au2–C49	2.022(7)	Ag3–C65	2.495(7)
Au3–C65	2.005(6)	Ag3–C113	2.420(6)
Au3–C81	1.973(6)	Ag3–C177	2.414(6)
Au4–C97	1.976(7)	Ag4–C49	2.393(7)
Au4–C113	1.972(7)	Ag4–C81	2.618(7)
Au5–C129	1.989(8)	Ag4–C209	2.605(7)
Au5–C145	2.025(9)	Ag4–C225	2.393(7)
Au6–C161	1.987(6)	Cu1–C1	1.938(10)
Au6–C177	1.994(7)	Cu1–C49	1.919(8)
Au7–C193	1.950(7)	Cu2–C17	2.018(8)
Au7–C209	2.000(7)	Cu2–C113	2.038(6)
Au8–C225	1.982(7)	Cu3–C129	1.941(9)
Au8–C241	1.979(7)	Cu3–C161	1.983(7)
Ag1–C33	2.364(6)	Cu4–C145	1.953(9)
Ag1–C161	2.422(6)	Cu4–C241	1.964(7)
Ag1–C193	2.558(8)		

Complex **4**

M–M distances (Å)

Au1–Au2	3.0862(6)	Au4–Au5	3.0704(6)
Au2–Au3	3.0439(5)	Au5–Au6	3.0467(5)
Au2–Cu1	3.0685(14)	Au5–Cu2	2.9981(12)
Au2–Cu3	3.0891(14)	Au5–Cu3	3.0847(11)
Au2–Cu6	3.0228(12)	Au5–Cu6	2.9438(16)
Au2–Cu5	2.9577(13)	Au5–Cu5	3.0012(10)
Au3–Cu3	2.9902(14)	Au6–Cu6	3.0348(15)

M–C(α) distances (Å)

Au1–C1B	1.979(8)	Cu2–C1B	2.025(8)
Au1–C27A	2.021(8)	Cu1–C1C	2.031(8)
Au2–C1K	2.068(7)	Cu1–C27A	1.947(10)
Au2–C1L	2.041(6)	Cu2–C1E	1.985(7)
Au3–C1C	1.963(8)	Cu3–C1D	1.969(8)
Au3–C1D	2.002(9)	Cu3–C1F	2.008(9)
Au4–C1G	1.954(9)	Cu4–C1G	1.999(9)
Au4–C1H	1.960(8)	Cu4–C1I	2.060(8)
Au5–C1E	2.029(6)	Cu5–C1L	2.025(8)
Au5–C1F	2.027(6)	Cu5–C1H	2.016(8)
Au6–C1I	1.948(7)	Cu6–C1J	1.995(8)
Au6–C1J	2.020(11)	Cu6–C1K	1.991(9)

Complex **5**

M–M distances (Å)

Au1–Ag2	3.1229(8)	Au5–Ag2	3.1139(9)
Au2–Ag1	2.8821(9)	Au5–Ag3	3.0978(8)
Au2–Ag2	2.9313(7)	Au5–Ag4	2.8841(9)
Au2–Ag3	2.9218(9)	Au5–Ag5	2.9070(7)
Au2–Ag5	3.0451(9)	Au5–Ag6	2.8969(11)
Au2–Ag6	3.1118(8)	Au6–Ag6	3.0631(10)
Au3–Ag3	3.0059(10)	Ag3–Ag6	3.1217(9)
Au4–Ag5	3.0941(9)		

M–C(α) distances (Å)

Au1–C1B	1.981(9)	Ag1–C1C	2.264(10)
Au1–C27A	1.966(8)	Ag1–C27A	2.196(10)
Au2–C1K	2.028(7)	Ag2–C1B	2.245(9)
Au2–C1L	2.020(8)	Ag2–C1E	2.300(8)
Au3–C1C	1.994(10)	Ag3–C1D	2.260(10)
Au3–C1D	2.005(10)	Ag3–C1F	2.265(10)
Au4–C1G	1.966(10)	Ag4–C1G	2.211(11)
Au4–C1H	1.998(10)	Ag4–C1I	2.291(11)
Au5–C1E	1.977(8)	Ag5–C1H	2.292(10)
Au5–C1F	2.005(7)	Ag5–C1L	2.262(9)
Au6–C1I	1.952(14)	Ag6–C1J	2.250(9)
Au6–C1J	2.036(12)	Ag6–C1K	2.320(9)

Complex **6**			
M–M distances (Å)			
Au1–Ag2(Cu2A)	3.0926(6)	Au4–Ag5(Cu5A)	3.0941(9)
Au2–Ag1(Cu1A)	2.8833(6)	Au5–Ag2(Cu2A)	3.1139(9)
Au2–Ag2(Cu2A)	2.9261(5)	Au5–Ag3(Cu3A)	3.0978(8)
Au2–Ag3(Cu3A)	3.0835(9)	Au5–Ag4(Cu4A)	2.8841(9)
Au2–Ag5(Cu5A)	2.9735(8)	Au5–Ag5(Cu5A)	2.9070(7)
Au2–Ag6(Cu6A)	3.0060(6)	Au5–Ag6(Cu6A)	2.8969(11)
Au3–Ag3(Cu3A)	3.0098(9)	Au6–Ag6(Cu6A)	3.0631(10)
M–C(α) distances (Å)			
Au1–C1B	2.018(6)	Ag1(Cu1A)–C1C	2.216(5)
Au1–C27A	2.006(6)	Ag1 (Cu1A)–C27A	2.159(7)
Au2–C1K	2.013(4)	Ag2(Cu2A)–C1B	2.168(6)
Au2–C1L	1.982(5)	Ag2(Cu2A)–C1E	2.203(5)
Au3–C1C	1.976(6)	Ag3(Cu3A)–C1D	2.032(6)
Au3–C1D	1.982(6)	Ag3(Cu3A)–C1F	2.073(6)
Au4–C1G	1.991(6)	Ag4(Cu4A)–C1G	2.142(6)
Au4–C1H	2.010(6)	Ag4(Cu4A)–C1I	2.203(5)
Au5–C1E	2.006(4)	Ag5(Cu5A)–C1H	2.098(6)
Au5–C1F	2.016(4)	Ag5(Cu5A)–C1L	2.107(5)
Au6–C1I	1.923(6)	Ag6(Cu6A)–C1J	2.093(5)
Au6–C1J	2.039(8)	Ag6(Cu6A)–C1K	2.158(5)

Complex **7**			
M–M distances (Å)			
Au1–Au1 [a]	3.2176(9)	Au4–Ag1	3.0570(18)
Au1–Ag1 [a]	2.7943(16)	Au4–Ag1A	3.050(3)
Au1–Ag1A [a]	2.853(3)	Au5–Ag1	2.9838(16)
Au2–Ag1A	3.128(3)		
M–C(α) distances (Å)			
Au1–C1	2.042(14)	Au5–C1	2.186(12)
Au1–C11	2.000(13)	Au5–C47	1.973(12)
Au2–C11	2.194(12)	Au3A–C37	2.214(15)
Au2–C21	2.001(14)	Au3A–C47	2.211(16)
Au3–C21	2.212(12)	Ag1–C37	2.300(14)
Au3–C27	2.199(11)	Ag1–C47	2.196(16)
Au4–C27	2.000(13)	Ag1A–C21	2.171(13)
Au4–C37	2.005(14)	Ag1A–C27	2.212(11)
Au4–C27	2.000(13)		

[a] 1-X,+Y,3/2-Z

Table S4 Photophysical data of **1**–**7**.

Complex	λ_{max} (nm)	τ (μs)	Φ
**1**	575	1.7	0.37
**2**	671	1.2	0.14
**3**	542	0.6	0.14
**4**	588	0.7	0.72
**5**	591	1.0	0.71
**6**	595	1.0	0.83
**7**	503	1.1	0.82

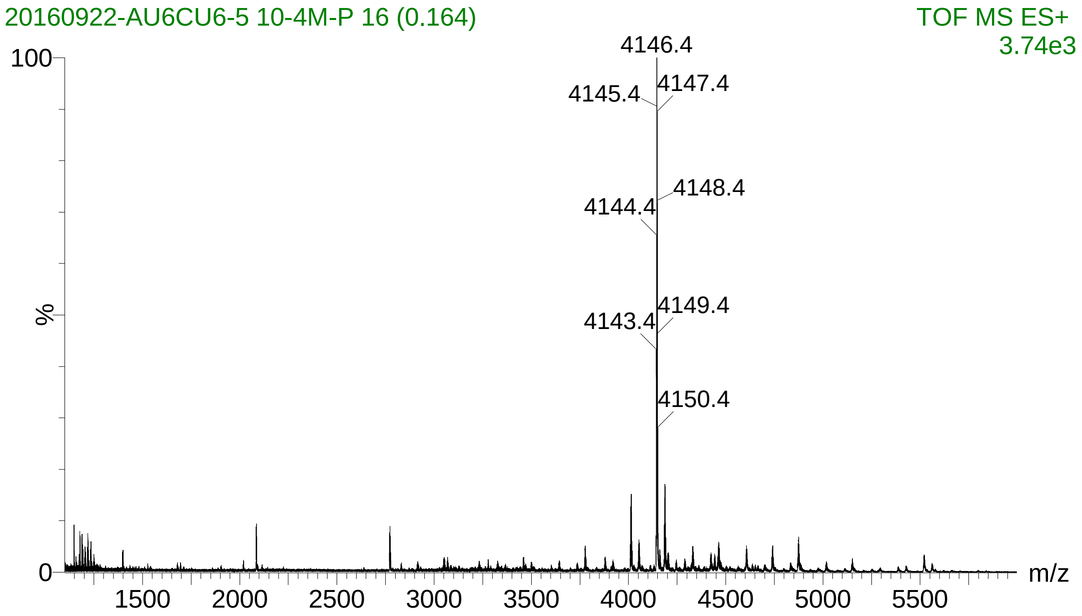

Fig. S1 ESI-MS spectrum of **1** in CH_2Cl_2/MeOH (c ≈ 10^{-4} M).

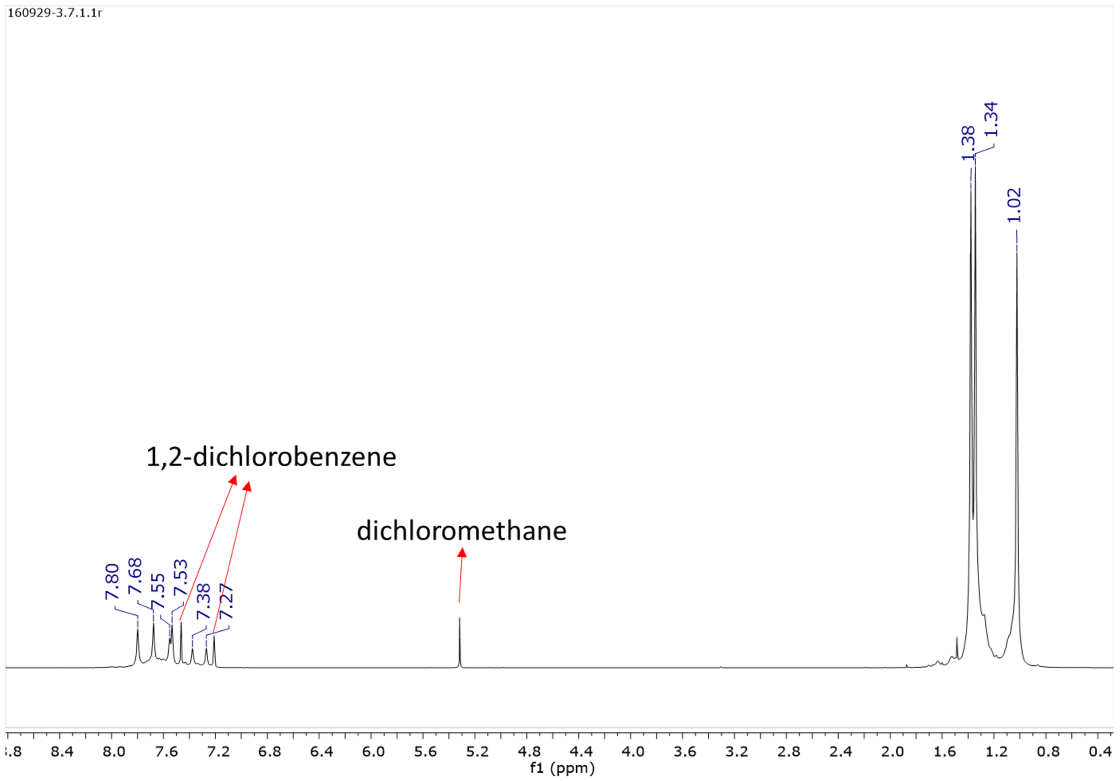

Fig. S2 ^{1}H NMR spectrum of **1** in 1,2-dichlorobenzene-d_4 at 298 K (dichloromethane was used as the solvent in the preparation and crystallization of **1**).

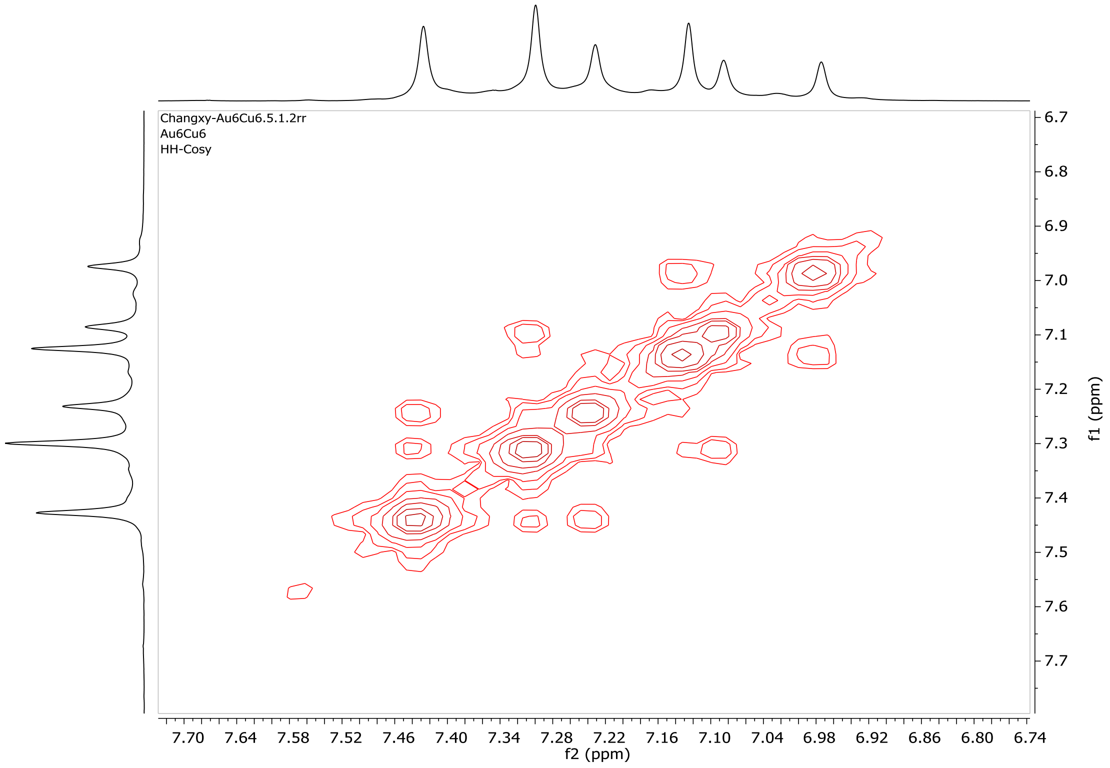

Fig. S3 ^{1}H-^{1}H COSY NMR spectrum (in aromatic region) of **1** in CD_2Cl_2 at 298 K.

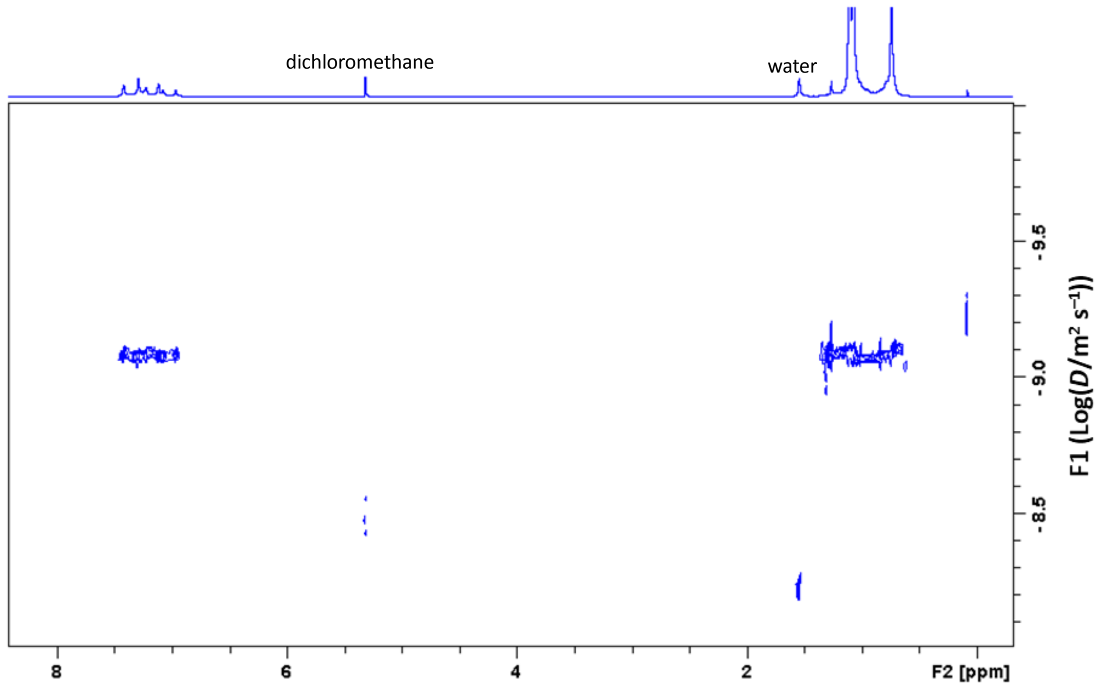

Fig. S4 ^{1}H DOSY NMR spectrum of **1** in CD_2Cl_2 at 298 K.

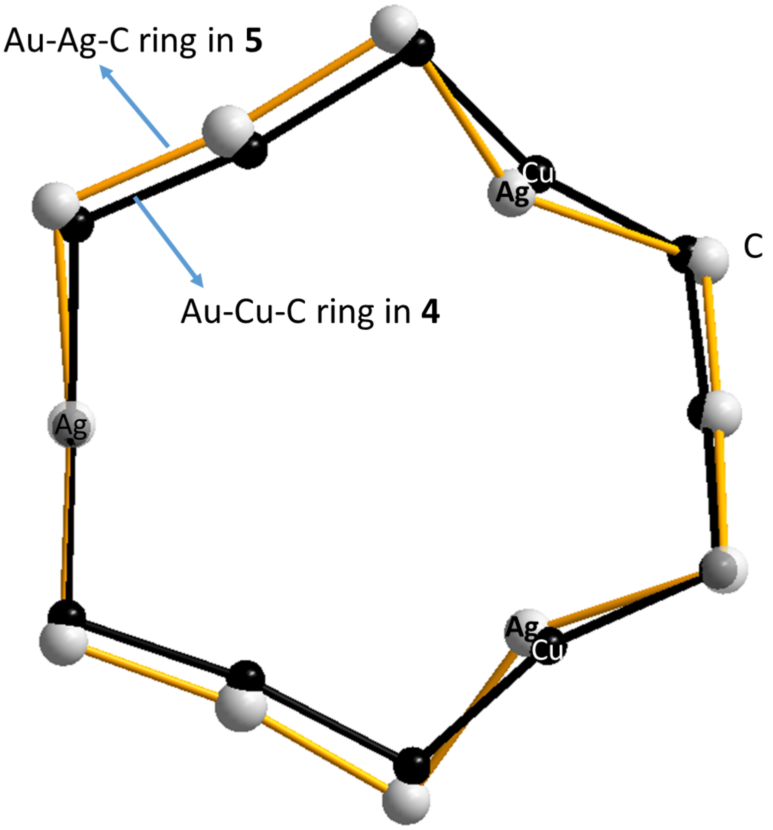

Fig. S5 Comparison of metallacycle core in **4** (white atoms and yellow bonds) and **5** (black atoms and black bonds).

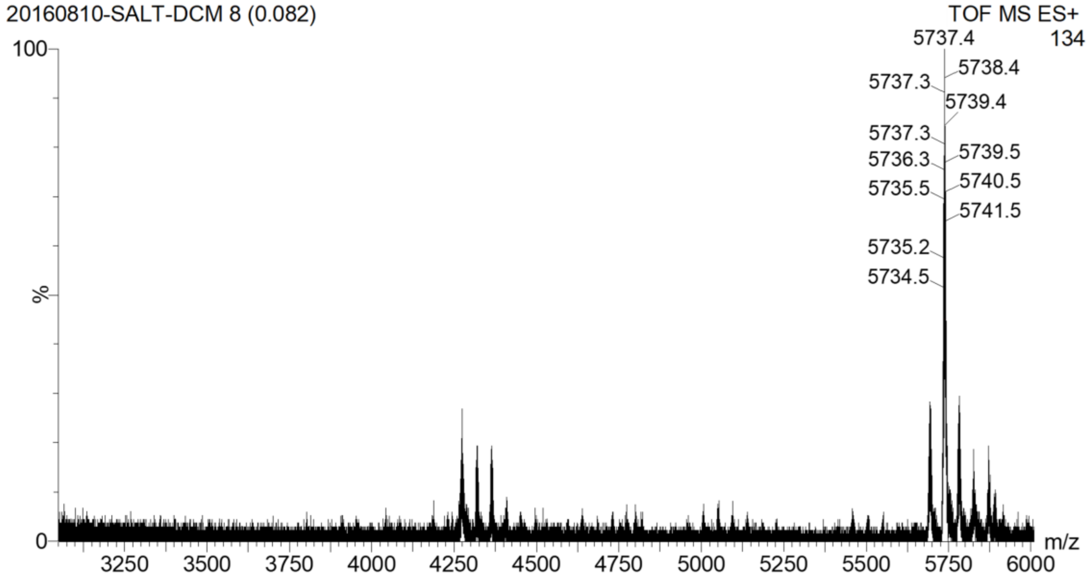

Fig. S6 ESI-MS spectrum of **3** in CH_2Cl_2.

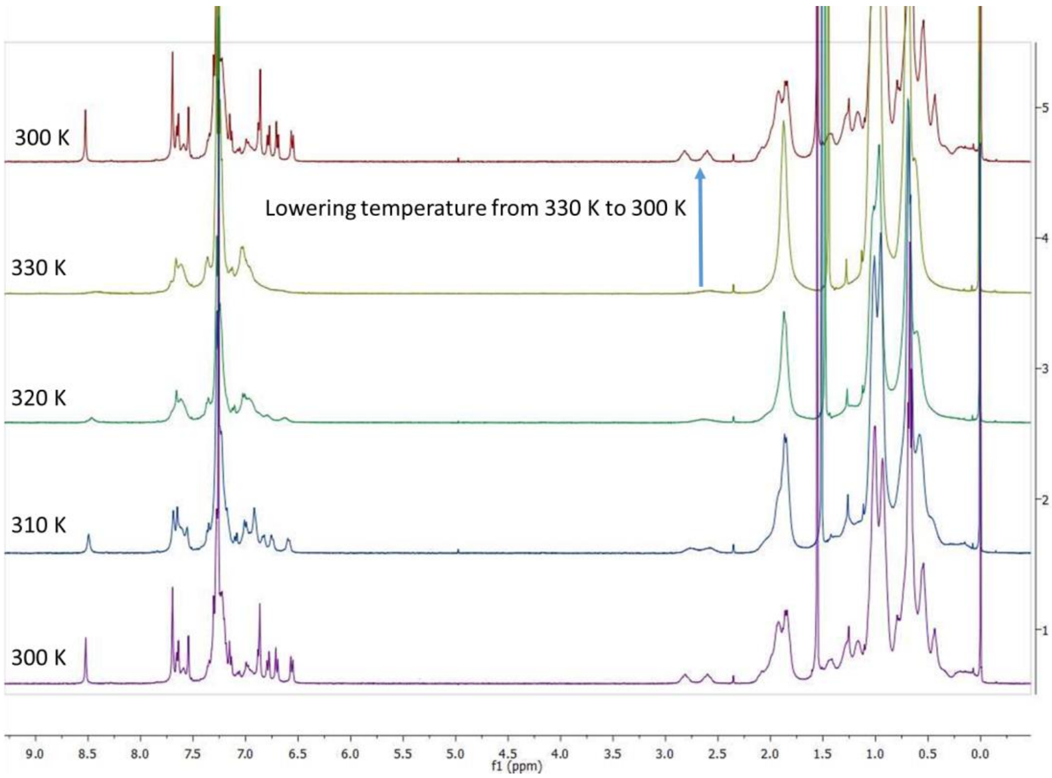

Fig. S7 Variable-temperature ^{1}H NMR spectra of **5** in $CDCl_3$ (300 K → 330 K → 300 K).

References

1 Y. Pan, Z. Peng and J. S. Melinger, *Tetrahedron*, 2003, **59**, 5495-5506.

2 K. A. Al-Farhan, M. H. Ja'far and O. M. Abu-Salah, *J. Organomet. Chem.*, 1999, **579**, 59-62.

3 *Proteum2*, Bruker AXS, Madson, Wisconsin, 1999.

4 G. M. Sheldrick, *Acta Crystallogr. Section A*, 2008, **64**, 112-122.

5 O. V. Dolomanov, L. J. Bourhis, R. J. Gildea, J. A. K. Howard and H. Puschmann, *J. Appl. Crystallogr.*, 2009, **42**, 339-341.

6 *R. D. Gaussian 09*, M. J. Frisch, G. W. Trucks, H. B. Schlegel, G. E. Scuseria, M. A. Robb, J. R. Cheeseman, G. Scalmani, V. Barone, B. Mennucci, G. A. Petersson, H. Nakatsuji, M. Caricato, X. Li, H. P. Hratchian, A. F. Izmaylov, J. Bloino, G. Zheng, J. L. Sonnenberg, M. Hada, M. Ehara, K. Toyota, R. Fukuda, J. Hasegawa, M. Ishida, T. Nakajima, Y. Honda, O. Kitao, H. Nakai, T. Vreven, J. A. Montgomery, Jr., J. E. Peralta, F. Ogliaro, M. Bearpark, J. J. Heyd, E. Brothers, K. N. Kudin, V. N. Staroverov, R. Kobayashi, J. Normand, K. Raghavachari, A. Rendell, J. C. Burant, S. S. Iyengar, J. Tomasi, M. Cossi, N. Rega, J. M. Millam, M. Klene, J. E. Knox, J. B. Cross, V. Bakken, C. Adamo, J. Jaramillo, R. Gomperts, R. E. Stratmann, O. Yazyev, A. J. Austin, R. Cammi, C. Pomelli, J. W. Ochterski, R. L. Martin, K. Morokuma, V. G. Zakrzewski, G. A. Voth, P. Salvador, J. J. Dannenberg, S. Dapprich, A. D. Daniels, Ö. Farkas, J. B. Foresman, J. V. Ortiz, J. Cioslowski and D. J. Fox, Gaussian, Inc., Wallingford CT, 2009.

7 J. P. Perdew, K. Burke and M. Ernzerhof, *Phys. Rev. Lett.*, 1996, **77**, 3865-3868.

8 J. P. Perdew, K. Burke and M. Ernzerhof, *Phys. Rev. Lett.*, 1997, **78**, 1396-1396.

9 S. Grimme, J. Antony, S. Ehrlich and H. Krieg, *J. Chem. Phys.*, 2010, **132**, 154104/1-154104/19.

10 P. C. Hariharan and J. A. Pople, *Theor. Chim. Acta*, 1973, **28**, 213-222.

11 M. M. Francl, W. J. Pietro, W. J. Hehre, J. S. Binkley, M. S. Gordon, D. J. DeFrees and J. A. Pople, *J. Chem. Phys.*, 1982, **77**, 3654-3665.

12 M. Kaupp, P. v. R. Schleyer, H. Stoll and H. Preuss, *J. Chem. Phys.*, 1991, **94**, 1360-1366.

13 A. Bergner, M. Dolg, W. Küchle, H. Stoll and H. Preuß, *Mol. Phys.*, 1993, **80**, 1431-1441.

14 M. Cossi, N. Rega, G. Scalmani and V. Barone, *J. Comput. Chem.*, 2003, **24**, 669-681.
